# Significance of Duodenal Prolactin Receptor Modulation by Calcium and Vitamin D in Sulpiride-Induced Hyperprolactinemia

**DOI:** 10.3390/medicina60060942

**Published:** 2024-06-04

**Authors:** Danijela Branislav Radojkovic, Milica Pesic, Milan Radojkovic, Marija Vukelic Nikolic, Tatjana Jevtovic Stoimenov, Sasa Radenkovic, Vojislav Ciric, Dijana Basic, Tatjana Radjenovic Petkovic

**Affiliations:** 1 Medical Faculty, University of Nis, Blvd. Dr Zoran Djindjic 81, 18000 Nis, Serbia; mimapesic@gmail.com (M.P.); radojkovici71@gmail.com (M.R.); marijavukelic@yahoo.com (M.V.N.); tjevtovic@yahoo.com (T.J.S.); doktor.sasa@gmail.com (S.R.); ciricv@yahoo.com (V.C.); 2 Clinic of Endocrinology, Diabetes and Metabolic Disorders, University Clinical Center Nis, Blvd. Dr Zoran Djindjic 48, 18000 Nis, Serbia; 3 Surgery Clinic, University Clinical Center Nis, Blvd. Dr Zoran Djindjic 48, 18000 Nis, Serbia; 4 Psychiatry Clinic, University Clinical Center Nis, Blvd. Dr Zoran Djindjic 48, 18000 Nis, Serbia; basicdijana321@gmail.com; 5 Center for Medical Biochemistry, University Clinical Center, Blvd. Dr Zoran Djindjic 48, 18000 Nis, Serbia; tatjanarp@gmail.com

**Keywords:** prolactin receptors, medicamentous hyperprolactinemia, vitamin D, calcium

## Abstract

*Background and Objectives:* Hyperprolactinemia, as a potential side-effect of some antipsychotic medications, is associated with decreased bone density and an increased risk of fractures. This study investigates whether calcium and vitamin D supplementation affects prolactin receptor (*Prlr)* gene expression in the duodenum, vertebrae, and kidneys of female rats with sulpiride-induced hyperprolactinemia. *Materials and Methods:* Twenty-one-week-old female Wistar rats were assigned to three groups: Group S consisted of ten rats who received sulpiride injections (10 mg/kg) twice daily for 6 weeks; Group D (10 rats) received daily supplementation of 50 mg calcium and 500 IU vitamin D along with sulpiride for the last 3 weeks; and Group C consisting of seven age-matched nulliparous rats serving as a control group. Real-time PCR was used to assess *Prlr* gene expression in the duodenum, vertebrae, and kidneys. *Results:* In Group S, Prlr gene expression was notably decreased in the duodenum (*p* < 0.01) but elevated in the vertebrae and kidneys compared to Group C. Conversely, Group D exhibited significantly increased Prlr expression in the duodenum (*p* < 0.01) alongside elevated expression in the vertebrae and kidneys. *Conclusions:* In sulpiride-induced hyperprolactinemia, decreased Prlr gene expression in the duodenum may lead to reduced intestinal calcium absorption. Consequently, prolactin may draw calcium from the skeletal system to maintain calcium balance, facilitated by increased Prlr gene expression in the vertebrae. However, vitamin D supplementation in sulpiride-induced hyperprolactinemia notably enhances Prlr gene expression in the duodenum, potentially ameliorating intestinal calcium absorption and mitigating adverse effects on bone health.

## 1. Introduction

Whenever we think about hyperprolactinemia (HP) and its negative impact on the skeletal system, the first thought is, “It has to be because of low estrogen”. Besides that fact, there is growing evidence about the direct role of prolactin on the bones. Prolactin receptors (*Prlr*), which are widely spread in the body [1], are also identified in the gastrointestinal tract [2] and bones [3,4,5], three key places for maintaining calcium homeostasis. Given that PRL has the ability to enhance active intestinal calcium absorption, stimulate bone remodeling, and decrease the excretion of calcium through the kidneys [6], it is considered one of the pivotal calciotropic hormones in the state of HP [7]. The literature data also indicate that different causes of hyperprolactinemia affect the skeletal system differently. During physiological HP (PHP), changes in maternal calcium homeostasis lead to decreased bone mineral density (BMD) [8], which is transient [9], completely recovered after weaning [5], and has no long-term detrimental effect on bone health [10,11]. On the other hand, drug-induced HP in various psychiatric disorders is considered a potential underlying reason for decreased BMD and increased risk of fractures [12,13,14]. Our previous experimental research highlighted the critical role of *Prlr* expression in organs involved in calcium metabolism. Notably, despite the similar duration of HP and PRL concentrations, significant differences were observed in *Prlr* gene expression in the intestine, kidneys, and bones between PHP and DIHP states. Increased *Prlr* expression in the intestine during PHP, facilitating calcium absorption, and decreased expression in the vertebrae, preventing calcium extraction from the skeletal system, may act protectively against maternal skeletal demineralization. Conversely, contrasting findings in DIHP, such as decreased *Prlr* expression in the intestine and increased expression in vertebrae, may contribute to the more severe effects on bone health associated with DIHP [15]. Unlike PHP, which typically has a limited duration, DIHP, as seen with certain antipsychotics, can persist for years. A significant decrease in BMD was found among schizophrenic patients on long-term use of antipsychotics and DIHP compared to drug-naive patients [16]. Despite the availability of several management options to mitigate the effects of antipsychotic-induced hyperprolactinemia, such as dose reduction, switching medications, or adding the partial agonist aripiprazole [17], it is frequently the case that the prolactin-elevating antipsychotic remains the only viable option to maintain stabilization of the patient’s condition. Although there is well-documented evidence linking antipsychotic use to hyperprolactinemia and its association with osteoporosis, there is a noticeable absence of preventive measures and treatment strategies to prevent osteoporosis in patients with DIHP.

We performed an experimental study to evaluate whether calcium and vitamin D supplementation affects *Prlr* gene expression in the duodenum, vertebrae, and kidneys of female rats with sulpiride-induced hyperprolactinemia.

## 2. Materials and Methods

### 2.1. Animals

Female Wistar rats, 21 weeks old, were provided by the Institute for Medical Research VMA (Military Academy of Medicine, Sofia, Bulgaria) in Belgrade, Serbia. The investigation took place at the Biomedical Research Centre, Medical Faculty, University of Nis, Serbia. Ethical approval for the experiment was obtained from the Ethical Committee of the Medical Faculty, University of Nis, Serbia (No. 01-2066-7).

The experimental animals had a weight range of 290–340 g. They were housed in a controlled environment with a 12:12 h light–dark cycle, with lights turning on at 06:00 AM. The rats were provided with standard chow and water. The room temperature was maintained at 23–25 °C, and the average humidity level was around 50–60%.

### 2.2. Experimental Design

Experimental animals were divided into the following groups:

Group S—sulpiride-induced hyperprolactinemia: 10 nulliparous rats (15 weeks old, plus 6-week intramuscularly administrated sulpiride (Eglonyl Alkaloid AD Skopje, Skopje, North Macedonia) (10 mg/kg) twice daily, which were 21 weeks old at the time of euthanasia). The aim of this experimental group was to investigate the expression of Prlr in the small intestine, spine, and reins under conditions of sulpiride-induced hyperprolactinemia.

Group D—vitamin D and calcium supplementation in sulpiride-induced hyperprolactinemia: 10 nulliparous rats (15 weeks old, plus 6-week intramuscularly administrated sulpiride (10 mg/kg) twice daily, calcium 50 mg daily and 1–2 drops daily, of oil solution Vigantol (MERCK D.O.O. Belgrade, Republic of Serbia) (500–1000 iU vitamin D) for last 3 weeks, which were 21 weeks old at the time of euthanasia). The aim of this animal group was to evaluate if there is a different expression of the *Prlr* gene in the duodenum, vertebrae, and kidneys in sulpiride-induced hyperprolactinemia with calcium and vitamin D supplementation.

Group C—control group: 7 age-matched nulliparous rats (21 weeks old) without previous pregnancy. The aim of this experimental group was to assess the relative expression of the Prlr gene in the small intestine, spine, and reins of rats between Groups S and C, as well as between Groups D and C, and to evaluate if there is any significant difference in expression during sulpiride-induced HP with and without calcium and vitamin D supplementation.

### 2.3. Laboratory Investigations

To obtain blood samples for laboratory measurements, a midline thoraco-abdominal incision was made, allowing access to the left myocardial ventricle in the rats, followed by euthanasia via exsanguination.

The concentration of PRL in the serum was measured using an enzyme-linked immunosorbent assay (ELISA) kit (Uscn Life Science Inc., Wuhan, China). The kit employed a sandwich enzyme immunoassay technique for quantitative measurements of PRL in rat serum, plasma, and other biological fluids, with a detection range of 3.12 to 200 pg/mL. The lowest detectable concentration of rat PRL was 1.03 pg/mL. The ELISA’s standard curve included concentrations of 200, 100, 50, 25, 12.5, 6.25, and 3.12 pg/mL. The intra-assay coefficient of variation (CV) was less than 10%. Additionally, the inter-assay CV was less than 12%, signifying good precision between different assays.

Vitamin D levels were determined using a 25-hydroxy vitamin D3 ELISA kit (CUSABIO), with a minimum detectable dose of rat vitamin D3 set at 5 μg/mL and a detection range of 20–100 μg/mL. The intra-assay coefficient of variation (CV) was less than 10%. Additionally, the inter-assay CV was less than 15%, signifying good precision between different assays.

Parathyroid hormone (PTH) levels were assessed using a rat PTH ELISA kit (CUSABIO). The detection range was 6.25–400 pg/mL, with a minimum detectable dose of rat PTH set at 1.56 pg/mL. The intra-assay CV was less than 8%. Similarly, the interassay CV was less than 10%, indicating good precision between different assays.

Serum-ionized calcium and inorganic phosphorus levels were analyzed in all experimental animals. Serum-ionized calcium levels were determined using the potentiometric method, while serum phosphate concentration was assessed through the photometric UV test utilizing the Beckman Coulter OLYMPUS analyzer (Brea, CA, USA). All experimental animals were individually housed in single-rat metabolic cages to collect 24 h urine samples for calciuresis and phosphouresis before euthanasia. A photometric color test was conducted to measure urine calcium levels, and a photometric UV test for urine phosphate concentration (Beckman Coulter OLYMPUS analyzer, Brea, CA, USA).

In all experimental animals, we conducted measurements of osteocalcin (OC) and serum procollagen type 1 N-terminal propeptide (P1NP).

The measurement of P1NP was conducted using an ELISA kit for P1NP (Uscn Life Science Inc.) with a minimum detectable concentration of rat P1NP of 10.7 pg/mL and a detection range of 31.2–2000 pg/mL. The concentrations used for the standard curve were 2000, 1000, 500, 250, 125, 62.5, and 31.2 pg/mL. The intra-assay coefficient of variation (CV) was less than 10%. Additionally, the inter-assay CV was less than 12%, signifying good precision between different assays.

N-MID osteocalcin kit (Cobas, Roche, IN, USA) with a detection range of 0.5–300 ng/mL, was used for determination of OC concentration. Values below the detection limit were indicated as “<0.5 ng/mL”. The intra-assay CV was 1.2–4.0%, and the inter-assay CV was less than 1.7% to 6.5%.

### 2.4. Tissue Preparation

Following the intramuscular administration of anesthesia (0.3 mL of 10% ketamine hydrochloride, an arylcyclohexylamine derivative (Ketamidor^®^, “Richter Pharma”), a midline thoraco-abdominal incision was performed.

We extracted a 10 cm long segment of the small intestine. To ensure cleanliness, we immersed the extracted segment in an ice-cold bath solution, after which a lengthwise cut was performed from the root of the mesentery, exposing the inner lining. Small intestine epithelial cells were collected by gently scraping the inner lining using an ice-cold glass slide [15,18]. The part of the lumbar spine L5-6 was removed and immersed in the ice-cold 0.1 M phosphate-buffered saline, pH 7.4, for the purpose of cleaning. At a temperature of 4 °C, cohesive connective tissues, muscles, and bone marrow were carefully eliminated from the samples, and skeletal tissue was promptly moved to liquid nitrogen used as cryogenic storage to facilitate subsequent fragmentation [4,15]. Collected renal tissue was rinsed in an ice-cold bath solution to remove any impurities and maintained at a temperature of −80 °C in an RNALater [15].

### 2.5. RNA Isolation and cDNA Synthesis

An aggregate of 20 mg of small intestine inner lining, lumbar spine, and renal tissue, which had been stored at −80 °C in RNALater, was used to isolate total RNA, following the manufacturer’s instructions using the RNeasy Mini Kit (Qiagen, Hilden, Germany). Quantification of the extracted RNA was performed using a QubitTM Fluorometer and the Qubit^®^ RNA Assay Kit (Invitrogen, Carlsbad, CA, USA). To ensure the removal of any potential DNA contamination, aliquots of RNA for qRT-PCR were treated with DNase I (Applied Biosystems, Waltham, MA, USA). Isolated RNA was then stored at −80 °C until further use, preserving its integrity and quality for subsequent analyses.

To generate cDNA from the total RNA samples (50 ng of each duodenum and kidney sample), a High-Capacity cDNA Reverse Transcription Kit (Applied Biosystems, Waltham, MA, USA) was utilized, in a total reaction volume of 20 μL, employing random hexamers (Applied Biosystems, Waltham, MA, USA) and an RNase inhibitor (Applied Biosystems, Waltham, MA, USA). The reactions were conducted utilizing a Mastercycler ep gradient S apparatus (Eppendorf, Hamburg, Germany). The temperature regimen for the reverse transcription process included an initial incubation at 25 °C for 10 min, followed by 120 min at 37 °C and 5 min at 85 °C. The resulting cDNA was stored at −80 °C until further use.

For the reverse transcription of the vertebra samples (a 20 ng total RNA of each vertebra sample), the Sensiscript Kit (Qiagen, Germany) was used. Random hexamers and an RNase inhibitor (both from Applied Biosystems, Waltham, MA, USA) were included in a total reaction volume of 20 μL. The temperature regimen for the reverse transcription process involved incubation at 37 °C for 60 min, followed by heating the samples to 95 °C for 5 min. The resulting cDNA was then stored at a temperature of −80 °C in a freezer until further use.

### 2.6. Relative Quantification of Prolactin Receptor Gene Expression Using RealTime PCR (RT-PCR)

For amplification of the *β2 microglobulin* gene (used as an endogenous control) and the target gene (long-form prolactin receptor), QuantiTect Primer Assays from Qiagen, Germany, were used (Table 1).

Amplification was tracked using a Stratagene Mx3005P system (Agilent Technologies, Santa Clara, CA, USA). QRT-PCR was conducted using 1 μL of cDNA, QuantiTect Primer Assay, and qPCR Master Mix-a (Kapa Biosystems, Wilmington, MA, USA) in a total reaction volume of 10 μL. Using RNA and water as negative controls allowed the detection of any background signal or false-positive results. The amplification reaction followed a thermal profile consisting of 3 min at 95 °C (for enzyme activation) followed by 40 cycles of amplification (each cycle included a 3 s DNA denaturation at 95 °C, a 20 s primer annealing at 55 °C, and a 1 s at 72 °C for elongation. To confirm the specificity of the amplification products, 2% agarose gel electrophoresis and melting curve analysis were performed. The acquired data were analyzed utilizing an instrument Stratagene Mx3005P, equipped with software MxPro 3005P (Agilent Technologies, Santa Clara, CA, USA).

### 2.7. Analysis of Real-Time PCR Results

The relative expression levels of mRNA for *Prlr* in the experimental Groups S and D were assessed by comparing them to the expression of the same mRNA in Group C, which served as the standard sample. To facilitate the comparison, the values of relative expression of mRNA for Prlr were determined as Log2.

### 2.8. Statistical Analyses

#### 2.8.1. Statistical Analyses of Laboratory Results

The results obtained from this study were analyzed using SPSS software, version 15.0. Continuous or measurable parameters were reported using descriptive statistics, including mean values (X) and standard deviation, as well as medians with the maximum and minimum values.

To determine the normality of parameter distribution, the Shapiro–Wilk test was employed. If the distribution of parameters was found to be normal, differences between groups were assessed using Student’s *t*-test for independent samples, and the Mann–Whitney U test was used if parameter distribution was deviated. The statistical analysis employed Student’s *t*-test for dependent samples in cases following a normal distribution, while the Wilcoxon test was utilized for assessing statistical significance between continuous parameter values at the beginning and end of this study in cases following a deviated distribution.

#### 2.8.2. Statistical Analyses of Real-Time PCR Results

The statistical analyses of real-time PCR data were conducted using the SPSS 15.0 software package. Within each experimental group, a statistically significant difference was determined using ANOVA analysis, which allowed the comparison of multiple groups simultaneously. The homogeneity of variance test was performed using Levene’s method. For subsequent post hoc analyses of multiple comparisons, either Tukey’s or Dunnett’s tests were used. The value of *p* ≤ 0.05 was considered statistically significant.

## 3. Results

### 3.1. Prolactin Levels

Levels of PRL were significantly higher in both experimental groups treated with sulpiride for six weeks, regardless of calcium and vitamin D supplementation, compared to the control group. There was no significant difference in PRL concentrations between experimental Groups S and D (Table 2).

### 3.2. Vitamin D Concentration in Experimental Groups

Vitamin D levels were significantly increased in D compared to C (96.34 ± 12.28 µg/mL vs. 49.12 ± 3.78 µg/mL; *p* < 0.001) and S (96.34 ± 12.28 µg/mL vs. 47.68 ± 2.96 µg/mL; *p* < 0.001). No significant difference was observed in vitamin D levels between experimental Groups S and C (47.68 ± 2.96 µg/mL vs. 49.12 ± 3.78 µg/mL; *p* > 0.05).

### 3.3. Parathyroid Hormone Levels

There was no significant difference in PTH concentrations between experimental groups (S vs. D: 145.13 ± 35.93 pg/mL vs. 168.87 ± 29.14 pg/mL; S vs. C: 145.13 ± 35.93 pg/mL vs. 158.11 ± 34.71 pg/mL; D vs. C: 168.87 ± 29.14 pg/mL vs. 158.11 ± 34.71 pg/mL; *p* > 0.05).

### 3.4. Mineral Analysis

Serum-ionized calcium concentration was within the reference range in all experimental groups. There was no significant difference in calcium concentrations between D and C. Phosphorus concentrations were not significantly changed among the experimental groups.

Total daily excretion of urinary calcium was significantly higher in D compared to S and C. The phosphorus excretion did not show a significant difference among the experimental groups (Table 2).

### 3.5. Bone Formation Markers

Osteocalcin concentration was significantly decreased in D compared to C, with no statistical significance between S and C (Table 2).

There was no significant difference in P1NP concentrations between experimental groups (Table 2).

### 3.6. Expression of Prlr mRNA in the Small Intestine in Experimental Groups S and D

Group D exhibited the highest relative expression of *Prlr.* Compared to Group C, Group D showed significantly higher *Prlr* mRNA expression, while Group S exhibited significantly lower *Prlr* expression (*p* < 0.01).

**Figure 1:**
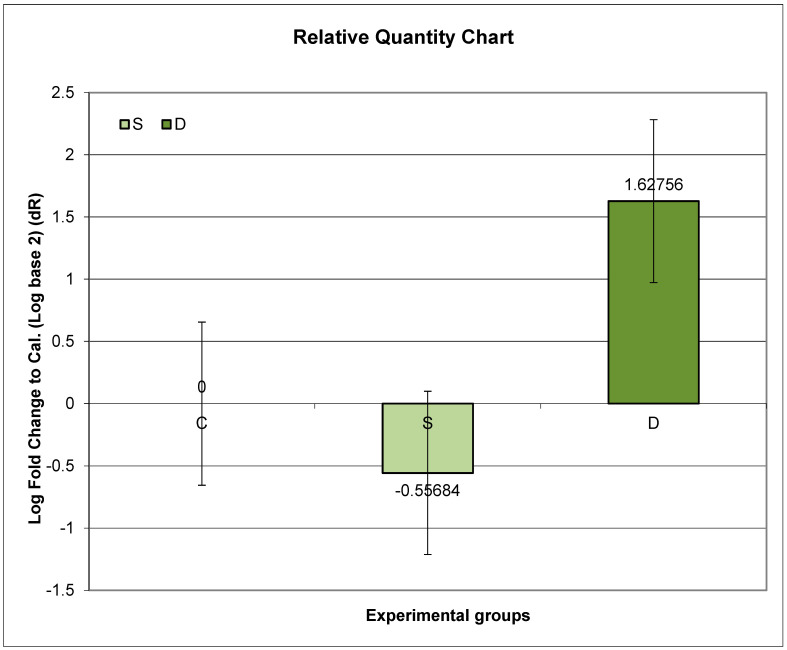
Relative expression of *Prlr* in the duodenum in sulpiride-induced hyperprolactinemia with and without vitamin D supplementation. Experimental groups: Group S—sulpiride-induced hyperprolactinemia; Group D—vitamin D and calcium supplementation in sulpiride-induced hyperprolactinemia; C—control group. Log2 (S/C) vs. (D/C) *p* < 0,01.

Further post hoc analyses unveiled that the ratio of relative *Prlr* mRNA expression between Groups D and C was significantly higher than the ratio between Groups S and C (*p* < 0.01) (Figure 1).

### 3.7. Expression of Prlr mRNA in Lumbar Spine in Experimental Groups S and D

Higher expression of *Prlr* mRNA was observed in Groups S and D, compared to C, but without statistical significance.

**Figure 2:**
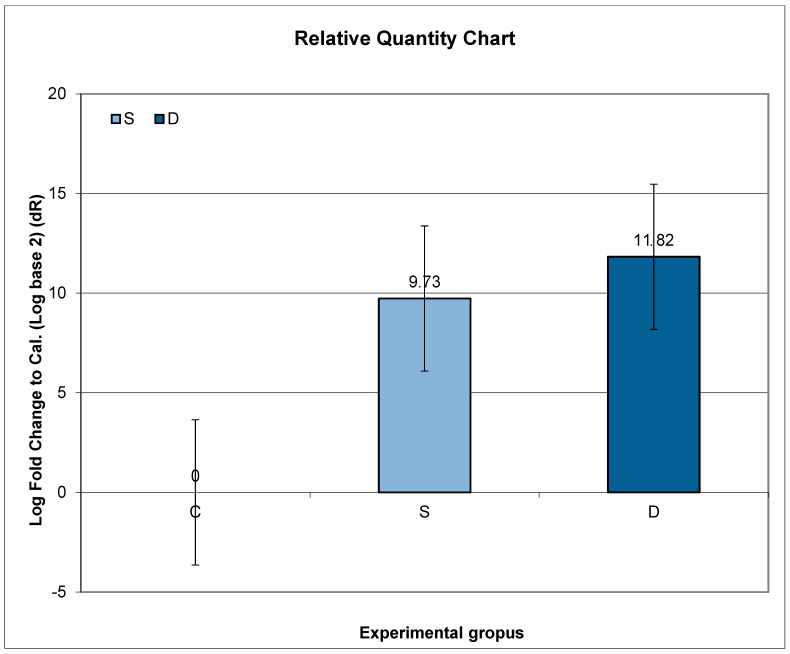
Relative expression of *Prlr* in the vertebrae in sulpiride-induced hyperprolactinemia with and without vitamin D supplementation. Experimental groups: Group S—sulpiride-induced hyperprolactinemia; Group D—vitamin D and calcium supplementation in sulpiride-induced hyperprolactinemia; C—control group. log_2_(S/C) vs. log_2_(D/C) (*p* > 0.05).

Further post hoc analyses indicated that the ratio of relative *Prlr* mRNA expression between Groups S and C did not exhibit a significant change compared to the ratio between Groups D and C (*p* = 0.1184; *p* > 0.05) (Figure 2).

### 3.8. Expression of Prlr mRNA in Renal Tissue in Experimental Groups S and D

The highest relative expression of *Prlr* in the kidney was in Group S. Even though higher expression of *Prlr* mRNA was observed in both experimental groups (S and D) compared to C, no statistical significance was found.

**Figure 3:**
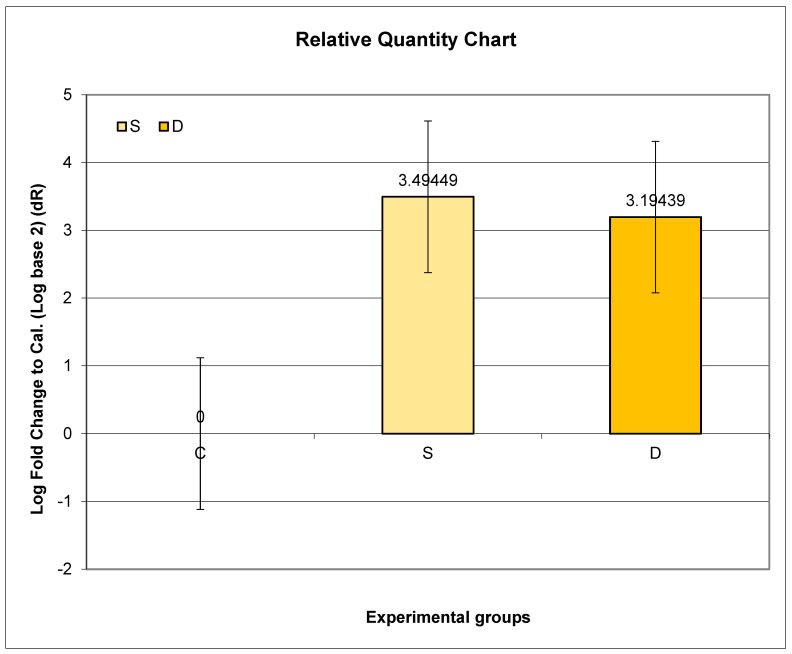
Relative expression of *Prlr* in the kidney in sulpiride-induced hyperprolactinemia with and without vitamin D supplementation. Experimental groups: Group S—sulpiride-induced hyperprolactinemia; Group D—vitamin D and calcium supplementation in sulpiride-induced hyperprolactinemia; C—control group. log_2_(S/C) vs. log_2_(D/C) (*p* > 0.05).

Further post hoc analyses indicated that the ratio of relative *Prlr* mRNA expression between Groups S and C did not exhibit a significant difference compared to the ratio between Groups D and C (*p* = 0.7317; *p* > 0.05) (Figure 3).

## 4. Discussion

### 4.1. Increased Prolactin Concentration in Experimental Groups

Prolactin (PRL) is a peptide hormone produced and released by lactotroph cells in the adenohypophysis. Dopamine is recognized as the major PRL-inhibiting factor. HP is a very frequent and important side effect of antipsychotic medication. The mechanism by which the majority of drugs induce hyperprolactinemia as a side effect is through the process of disinhibition. Since the pituitary gland is localized outside the blood–brain barrier, antipsychotic drugs with a high ability to cross this barrier can easily reach the pituitary, block D2 receptors on lactotroph cells, remove the inhibitory effect of dopamine, and free the pituitary gland to spontaneously secrete prolactin at a very high rate [19,20].

Sulpiride is a second-generation antipsychotic medication designed to achieve a more potent blockade of dopamine receptors [20]. In addition to its use as an antipsychotic, sulpiride is also prescribed as an antidopaminergic gastrointestinal prokinetic agent. The prokinetic effect of sulpiride is achieved through the blockade of enteric D2 receptors, and the antiemetic effect is the result of blocking central D2 receptors. The use of sulpiride is often followed by increased PRL levels, which are maintained during the entire time of therapy [18,19,20,21,22].

This study’s results verified significantly increased PRL in sulpiride-treated subjects, which was considered medicamentous hyperprolactinemia.

### 4.2. Changes in Mineral Analysis during Sulpiride-Induced HP

#### 4.2.1. Serum-Ionized Calcium and Phosphorous

During the 6-week sulpiride therapy, calcium concentrations remained within the normal reference range. Most studies in the literature have reported normal calcium levels during antipsychotic therapy [14,15,23,24,25,26], with only a few cases of hypocalcemia [27,28,29]. Even when calcium levels decreased compared to baseline, they still remained within the normal range, as seen in this study. Therefore, a more accurate term for the relationship between antipsychotics and calcium would be “fluctuation” rather than hypo- or hypercalcemia.

Serum phosphate levels generally remain normal during physiological and medicamentous hyperprolactinemia in humans and animals [14,15,29]. The minor variations observed in serum phosphorus concentrations can be attributed to the higher efficiency of dietary phosphorus absorption compared to dietary calcium absorption [30].

#### 4.2.2. Urinary Calcium and Phosphorus Excretion

This study’s results did not verify significant alterations in urinary calcium and phosphorus excretion during the 6-week sulpiride-induced HP. During calcium and vitamin D supplementation, we observed a significant increase in urinary calcium excretion, which is consistent with previous studies [31,32].

### 4.3. Changes in Bone Turnover Markers during Sulpiride-Induced HP

Osteocalcin (OC) and procollagen type 1 N-terminal propeptide (P1NP) are reliable markers of bone turnover, reflecting different aspects of osteoblastic activity. OC primarily indicates bone turnover during mineralization, while P1NP specifically reflects osteoblast proliferation and collagen synthesis [33].

OC synthesized by osteoblasts binds calcium from the blood circulation and transports it to the bone matrix. However, OC is initially inactive and requires vitamin K2-7 for conversion into its active form through carboxylation. Vitamin K2-7 serves as a cofactor for the enzyme γ-carboxylase, which converts glutamic acid (Glu) residues within OC molecules to γ-carboxyglutamate (Gla), thus facilitating the γ-carboxylation process of OC [34,35,36].

The literature data regarding OC levels in drug-induced HP are contradictory, with some studies reporting increased levels in certain conditions [28,37] and others showing decreased levels [15,38,39]. In this study, sulpiride-induced HP with calcium and vitamin D supplementation resulted in a significant decrease in OC levels. The effects of vitamin D therapy on OC levels have shown conflicting results in previous studies, with some reporting an increase, which was interpreted as an improvement in osteosynthesis [37,40], and others demonstrating a decrease [41,42]. Literature on vitamin K levels in patients with drug-induced hyperprolactinemia is currently lacking. It could only be hypothesized that low levels of osteocalcin (OC) may be attributed to insufficient vitamin K2-7 in this population.

Limited data exist on P1NP levels in hyperprolactinemia. In our previous study, we observed increased P1NP levels in physiological hyperprolactinemia during late pregnancy and decreased levels in sulpiride-induced hyperprolactinemia [15]. However, in this study, P1NP concentrations did not show significant changes during 6-week sulpiride-induced hyperprolactinemia, regardless of calcium and vitamin D supplementation. Interpretations of changes in bone synthesis markers like OC and P1NP are not always consistent, as a decrease in these markers could indicate a cessation of bone resorption [43].

### 4.4. Analysis of Prlr Expression in the Duodenum during Sulpiride-Induced HP with and without Vitamin D Supplementation

Under physiological conditions, intestinal calcium absorption occurs through both passive paracellular and active transcellular routes. The active route involves specific calcium channels for entry, intracellular transfer, and extrusion via the calcium pump [44]. Vitamin D plays a crucial role in regulating active intestinal calcium absorption through its receptor (VDR) [44,45,46]. In addition to vitamin D, there is growing evidence of direct PRL influence on intestinal calcium absorption. Experimental studies with PRL, including endogenous and exogenous PRL [47,48,49,50], with pituitary graft [51,52], as well as conditions like pregnancy and lactation [6,15], have demonstrated PRL’s significant role in both active and passive intestinal calcium absorption [53,54,55,56].

This study found significantly decreased *Prlr* expression in the duodenum during 6-week sulpiride-induced HP, which is consistent with our previous findings of a shorter duration of medicamentous HP [15] and quite opposite to increased *Prlr* expression in physiological HP as pregnancy and lactation [15,55]. The process by which DIHP downregulates *Prlr* expression in the duodenum, while PHP upregulates *Prlr* in the same tissue, is not fully understood. One possible explanation could be that sulpiride has a direct effect on the digestive tract, contributing to this phenomenon. On the other hand, PHP may trigger different signaling cascades that lead to the upregulation of *Prlr* in the same tissue. Further research is needed to elucidate the underlying mechanisms and clarify the observed differences. There are no literature data, to our knowledge, about prolonged medicamentous HP’s effect on *Prlr* expression in the duodenum. The downregulation of *Prlr* in the duodenum during medicamentous HP, verified in this study, may reduce PRL’s role in intestinal calcium absorption [47,48,49,50,51,52,53,54,55,56]. Previous investigations revealed that PRL has a direct stimulatory effect on vitamin D production [57,58]. Van Cromphaut et al. [57] demonstrated that pregnancy and lactation equally enhance transient receptor potential vanilloid type 6 (TRPV6) expression in the duodenum in both wild-type and VDR-null mutant mice. This suggests that there are vitamin-D-independent effects on duodenal calcium absorption mechanisms during pregnancy and lactation. In the experimental study conducted by Ajibade et al. [58], vitamin-D-deficient mice were used and treated with prolactin alone or prolactin and 1,25(OH)_2_D_3_ to determine whether prolactin may upregulate intestinal vitamin D target genes and whether this regulation is dependent or independent of 1,25(OH)_2_D_3_. Their findings indicated that prolactin can regulate TRPV6 and the 1α(OH)ase gene and that prolactin has cooperative effects with 1,25(OH)_2_D_3_ in regulating intestinal calcium transport proteins, as well as intestinal calcium transport. This study’s results further showed that adding calcium and vitamin D supplementation significantly increased *Prlr* expression in the duodenum, potentially improving calcium absorption. Such a synergism of prolactin and vitamin D in calcium intestinal absorption suggests the therapeutic potential of vitamin D in medicamentous HP.

### 4.5. Analysis of Prlr Expression in the Vertebrae during Sulpiride-Induced HP with and without Vitamin D Supplementation

Hyperprolactinemia is a well-known risk factor for decreased bone mass. While there is evidence supporting the direct role of prolactin in skeletal metabolism, the prevailing belief is still focused on prolactin-mediated hypogonadism. In vitro studies have confirmed that prolactin suppresses osteoblast proliferation and reduces overall osteoblast numbers in bone tissue [59]. There is also evidence of prolactin’s impact on reduced bone mineralization [59,60]. The contradictory nature of having osteoblasts as the primary target and increased osteoclast activity is yet to be confirmed, as direct effects of prolactin on osteoclasts are not established. In hyperprolactinemia, resorptive activity is predominant due to decreased bone volume and trabecular number [59].

This study found increased *Prlr* expression in the vertebrae during 6-week sulpiride-induced HP, consistent with our previous findings [15]. This upregulation suggests a heightened influence of PRL on bone metabolism, potentially leading to slower osteoblast differentiation and reduced osteoblast number [59]. It may serve as a compensatory mechanism to decrease intestinal calcium absorption, simultaneously causing more detrimental effects on bone metabolism during medicamentous HP.

In the group treated with calcium and vitamin D, alongside 6-week sulpiride-induced hyperprolactinemia, there was a non-significant decrease in *Prlr* mRNA expression. The direct role of vitamin D in bone formation is still debated, with conflicting views on whether its effects are solely indirect through regulating calcium levels in the intestines and kidneys or also direct on osteoblasts. However, the expression of vitamin D receptor (VDR) in osteoblasts suggests a direct influence. Vitamin D has been shown to stimulate bone formation and mineralization in studies using human osteoblasts [61,62,63]. Recent findings indicate that vitamin D induces mineralization during the pre-mineralization period [64].

Given that both hormones impact osteoblasts, with PRL reducing their proliferation and total number and vitamin D influencing their growth and differentiation, longer vitamin D administration may mitigate the harmful effects of hyperprolactinemia on the skeletal system.

### 4.6. Analysis of Prlr Expression in the Kidney during Sulpiride-Induced HP with and without Vitamin D Supplementation

The role of Prlr in the kidney is not fully understood despite various studies on its expression [1,65,66]. Prolactin has been implicated as a natriuretic hormone by inhibiting Na(+)/K(+)-ATPase activity in the renal tubules [66,67]. In this study, increased *Prlr* expression in the kidney was observed during sulpiride-induced HP. The addition of calcium and vitamin D showed a non-significant decrease in *Prlr* expression. Based on limited data regarding PRL’s role in renal tissue, it can only be postulated that increased *Prlr* expression in HP may lead to increased natriuresis and indirect calcium elimination. Further research is needed to understand the precise mechanisms of PRL’s influence on calcium excretion.

This study has several limitations. With regret, we acknowledge that our institution was lacking the following capabilities: measurement of bone resorption markers, determination of the total concentration of 25(OH)D (25-hydroxyergocalciferol and 25-hydroxycholecalciferol), measurement of serum vitamin K2 concentrations, and assessment of direct intestinal calcium absorption rate. Given that the results were obtained from an animal experimental model, the absence of a group of patients with drug-induced hyperprolactinemia could also be considered as a limitation. We highlight this limitation as a constraint in this study.

## 5. Conclusions

Decreased *Prlr* expression in the duodenum during sulpiride-induced hyperprolactinemia may contribute to reduced intestinal calcium absorption. To maintain normal calcium levels, PRL may target other tissues like the kidney and skeletal system. Increased *Prlr* expression in vertebrae allows PRL to extract calcium from bones, potentially leading to bone density depletion and increased fracture risk. Vitamin D significantly increased *Prlr* expression in the duodenum, suggesting its therapeutic potential in medicamentous hyperprolactinemia by enhancing intestinal calcium absorption.

## Figures and Tables

**Table 1:** Commercial RNA-specific primers used in this experiment.

QuantiTect Primer Assay (Qiagen, Germany)		Detected Transcript
β2 microglobulin	Rn_B2m_1_SG, QT00176295	NM_012512
Prolactin receptor gene	Rn_Prlr_vb.1_SG, QT01169518	NM_001034111

**Table 2:** Laboratory analysis of the experimental groups.

	S	D	C
PRL (pg/mL) X ± SD	148.92 ± 20.46 ^a^	148.38 ± 27.25 ^b^	112.01 ± 11.92
s-Ca ^++^ (mmol/L) X ± SD	1.21 ± 0.03	1.17 ± 0.03	1.15 ± 0.02
s-P (mmol/L) X ± SD	1.70 ± 0.13	1.92 ± 0.16	1.89 ± 1.89
u-Ca (mmol/24 h) X ± SD	2.88 ± 0.60	8.63 ± 2.08 ^c,d^	3.37 ± 0.87
u-P (mmol/24 h) X ± SD	53.93 ± 14.05	67.83 ± 16.78	55.03 ± 20.37
OC (ng/mL) X ± SD	13.55 ± 3.42	9.90 ± 1.98 ^e,f^	16.18 ± 2.00
P1NP (pg/mL) X ± SD	291.70 ± 71.03	283.70 ± 86.52	314.86 ± 50.99

Experimental groups: Group S—sulpiride-induced hyperprolactinemia; Group D—vitamin D and calcium supplementation in sulpiride-induced hyperprolactinemia; C—control group. PRL—prolactin; s-Ca^++^—serum calcium concentration; s-P—serum phosphorus concentration; u-Ca—urine calcium concentration; u-P—urine phosphorus concentration; OC—osteocalcin; P1NP—procollagen type 1 N-terminal propeptide. ^a^ S vs. C, *p* < 0.001; ^b^ D vs. C, *p* < 0.01; ^c^ D vs. C, *p* < 0.001; ^d^ D vs. S, *p* < 0.001; ^e^ D vs. C, *p* < 0.001; ^f^ D vs. S, *p* < 0.01.

## Data Availability

The original contributions presented in the study are included in the article. Further inquiries can be directed to the corresponding author/s.
